# Microcognitive science: bridging experiential and neuronal microdynamics

**DOI:** 10.3389/fnhum.2013.00617

**Published:** 2013-09-27

**Authors:** Claire Petitmengin, Jean-Philippe Lachaux

**Affiliations:** ^1^Télécom École de Management, Institut Mines-TélécomEvry, France; ^2^Archives Husserl, École Normale SupérieureParis, France; ^3^Brain Dynamics and Cognition Team, Lyon Neuroscience Research Center, INSERM, U1028, CNRS, UMR 5292Lyon F-6950, France; ^4^University Lyon 1Lyon F-69500, France

**Keywords:** elicitation, neuro-phenomenology, microcognition, microdynamics, neurofeedback, enaction, microgenesis, gamma band

## Abstract

Neurophenomenology, as an attempt to combine and mutually enlighten neural and experiential descriptions of cognitive processes, has met practical difficulties which have limited its implementation into actual research projects. The main difficulty seems to be the disparity of the levels of description: while neurophenomenology strongly emphasizes the micro-dynamics of experience, at the level of brief mental events with very specific content, most neural measures have much coarser functional selectivity, because they mix functionally heterogeneous neural processes either in space or in time. We propose a new starting point for this neurophenomenology, based on (a) the recent development of human intra-cerebral EEG (iEEG) research to highlight the neural micro-dynamics of human cognition, with millimetric and millisecond precision and (b) a disciplined access to the experiential micro-dynamics, through specific elicitation techniques. This lays the foundation for a microcognitive science, the practical implementation of neurophenomenology to combine the neural and experiential investigations of human cognition at the subsecond level.

Despite the immediate appeal of neurophenomenology, as an attempt to combine and mutually enlighten neural and experiential descriptions of cognitive processes (Varela, 1996), practical difficulties have limited its implementation into actual research projects. In spite of some success (Lutz et al., 2002; Petitmengin et al., 2007), an acknowledged challenge has been to find a level of observation at which convergence is possible. Neurophenomenology strongly emphasizes the micro-dynamics of experience, at the level of brief mental events with very specific content, such as visual perception or access to the meaning of a word. However, most neural measures have much coarser functional selectivity, because they mix functionally heterogeneous neural processes either in space (i.e., scalp-level EEG) or in time (i.e., fMRI). Such discrepancy makes it extremely difficult to even try a correlation between neural and experiential descriptions. In this paper, we propose a new starting point for neurophenomenology, based on (a) the recent development of human intra-cerebral EEG (iEEG) research to reveal the neural micro-dynamics of human cognition, with millimetric and millisecond precision and (b) a disciplined access to the *experiential* micro-dynamics, through specific elicitation techniques. If a bridge is to be built between the neural and experiential levels, it should be done where the river is shallow, where descriptions of mental processes are fine-grained on both sides. This means simple cognitive operations, at the level of the perception-action cycle (Fuster, 2004; Madl et al., 2011), and precise measures of their micro-dynamics. This lays the foundation for a micro-cognitive science: the practical implementation of neurophenomenology to combine the neural and experiential investigations of human cognition at the subsecond level.

## Eliciting experiential microdynamics

In most cognitive “tasks” we might be involved in, whether it is reading an article, listening to a piece of music or cooking a cake, our attention is usually almost completely absorbed into the content, the “what” of our activity, to the detriment of the “how.” For example, when hearing a sound, we immediately connect that sound to a possible physical cause: “It's a blackbird in the plum tree,” without noticing the rapid micro-operations we realize in order to recognize that sound (Ihde, 1976/2007; Petitmengin et al., 2009). While writing this article, my attention is absorbed in the content of the ideas I am trying to express, but I am hardly aware of the rapid succession of inner comments, light emotions, evaluations, and comparisons that I realize instant after instant to find the right words and evaluate their appropriateness. While reading this sentence, are you aware of the rapid sequence of micro-operations that enable you to go from the words to their meaning, paced by several saccadic eye-movements each second? As the well-known experiments of Nisbett and Wilson clearly showed, even our decision processes largely elude our awareness (Nisbett and Wilson, 1977). This blindness to the dynamics of experience seems to affect most, if not all our cognitive processes, from the most concrete to the most abstract ones.

However, recent studies show that this lack of awareness is not a fatality. By carrying out specific mental acts, we can access our cognitive processes and describe them with reliability and great precision (Petitmengin et al., 2013).

The first condition to this access is to shift from a general description to the description of a particular occurrence of the cognitive process to be described, a singular experience which is precisely situated in time and space.

The second condition for accessing experiential dynamics consists in loosening the focus of attention on the “what” of the process in order to let the “how” appear. For example, if a mental image is emerging into our consciousness, we have to reorient our attention from the content of the image (for example a blooming cherry tree), toward the dynamics of appearance, the genesis of this content: the rapid phases which precede its stabilization, and at each phase, the subtle inner micro-gestures that we perform to elicit, stabilize, recognize, evaluate, rule out, or enrich this image. Becoming aware of this microdynamics requires a particular mode of attention, which is both unfocused, diffuse, receptive, and very acute, sensitive to the most subtle discontinuities. Performing this reorientation of attention is a skill which can be learnt. This inner act may also be triggered by an expert interviewer, in the context of an “elicitation interview,” through relevant prompts and questions (Vermersch, 1994/2011; Petitmengin, 2006). The structure of an elicitation interview is an iterative structure which consists of helping subjects to evoke the experience to be described several times, while guiding their attention toward a diachronic mesh which is finer each time, until the required level of detail is reached. For example, after encouraging an epileptic patient to retrieve the circumstances of her last seizure, I led her to describe in detail the sequence of this experience. Here is a short excerpt of interview:

- At that point I get off my bike.
- Why do you get off your bike?
- (Silence …) I know that I'm about to have a seizure.
- Take the time to go back to that moment… At that moment, how do you know that you're about to have a seizure?
- (Silence…) Because I feel this sensation in my chest…
- Because you feel this sensation in your chest. When you feel this sensation, what do you feel?
- (Silence…) It's a sensation of compression.
- I would like you to go back to the moment when you are still on your bike, before getting off. How does this sensation of compression appear?
- (Silence…) In fact, progressively. It rises from my stomach to my head.
- Could you come back to the moment when the sensation is at the level of your stomach? When does it appear exactly? How is it like? How big is it?

And so on. The same type of questioning can be used to obtain the microdynamic description of actions, whether physical actions or inner acts. For example, here is a brief extract of the interview of a meditator:

- At that moment I am in a kind of availability, of expectation, and also a kind of curiosity, lucidity, I am present …
- How do you know that you are present?
- I am not in my head anymore, I am listening with my whole body.
- How do you go about listening with your whole body?
- First, I'm going to place my consciousness much more toward the back of my skull.
- How do you go about placing your consciousness toward the back of your skull?

This interview method has been used extensively to collect detailed descriptions of unnoticed microdynamics in many cognitive and clinical domains. For example loosening the attentional focus on the content of an idea (for example a scientific idea concept) makes it possible to discover a process of maturation, and micro-adjustments of attention facilitating the progressive transformation of a fuzzy and blurred feeling into a “clear and distinct” idea (Petitmengin, 1999, 2007; Remillieux, in press): in other words, an invisible microgenesis, of which the idea is only the visible result. Elicitation methods also allow precise descriptions of the microdynamics of decision processes, usually concealed by the absorption of attention into the outcome of the process (Petitmengin et al., 2013). In the clinical domain, in-depth elicitation interviews have enabled epileptic patients to become aware of early signs announcing the arrival of an epileptic seizure, although seizures were considered previously as unpredictable. This confirms on the experiential level what has already been discovered on the neuronal level (Martinerie et al., 1998): that seizures do not arise “like a bolt in the blue,” but are the visible result of a process that has started long before. Importantly, the awareness of this microgenesis is the key to new therapies for epilepsy, whose results are often better than those of the most efficient pharmacological treatments (Petitmengin et al., 2006): this clearly highlights the therapeutic stake of becoming aware of the microdynamics of lived experience.

Elicitation methods allow very fine-grained descriptions of the experiential microdynamics: subtle adjustments of the orientation, scope, source, and intensity of attention, rapid succession of micro-operations of comparison, evaluation, verification, fine variations of emotional states, and so on. This work makes it possible to detect the dynamics of appearance of phenomenal dimensions which are usually experienced as an inseparable whole, such as the different sensorial dimensions (visual, auditory, tactile, and olfactory) of an imagined scene or a perceptive illusion. For example, while the phenomenological descriptions of the well-known “rubber hand illusion” usually focus on the final illusion (a rubber hand is felt as if it was one's own hand), elicitation techniques enable the description of the genesis of this illusion (Valenzuela et al., 2013). With the help of these techniques, several subjects described the successive transfer from the real hand to the rubber hand of two dimensions of tactile experience which are usually felt as inseparable, the “touching” and the “touched” dimensions (Merleau-Ponty, 1945). A careful work of elicitation allows access to even earlier phases of the microgenetic process, which seem to share common characteristics (Petitmengin, 2007):

- fuzzy feelings which do not fall within a particular sensorial modality, but have “transmodal” submodalities (such as intensity and rhythm)—submodalities which are not specific to a particular sense, but transposable from one sense to another^1^.
- an alteration of the sense of agency, under the form of an absence of control over the cognitive event which emerges;
- an alteration of the sense of ownership: the idea, the sensation… is not felt as being immediately *mine*, it is not felt as *personal*.

The “temporal granularity” of microgenetic descriptions is also very fine. For example, we collected detailed descriptions of the various micro-actions involved in choice processes lasting for 1 or 2 s (Petitmengin et al., 2013), or in auditory perceptions lasting for less than a second (Petitmengin et al., 2009). We estimate that elicitation techniques provide access to micro-actions of one-quarter of a second, which is the typical duration of a perception-action cycle (see Jung et al., 2008, for a measure of that duration during reading).

Once microdescriptions of singular experiences have been collected, the second part of the work consists in analyzing them in order to identify possible regularities. Each time we analyzed a corpus of experiential descriptions, even if the *content* of the experiences was very different, we were able to detect generic microdynamic *structures* (Petitmengin et al., 2009, 2013). For example, even if the content of the ideas which we explored the genesis of was very different, we discovered a generic structure of this genesis (Petitmengin, 1999, 2007). The very existence of these structures has an important epistemological consequence: it enables the production of results which are reproducible and therefore verifiable.

## Investigating neuronal microdynamics

The development of experiential microdynamics has been paralleled by recent advances in the study of the neural microdynamics supporting human cognition. At the end of the 1990's, two research groups (Crone et al., 1998; Lachaux et al., 2000) claimed that neural processing underlying cognition could be mapped with very high spatial and temporal precision, and very high signal-to-noise ratio, from the high-frequency components of intracerebral EEG recordings obtained in the brain of epileptic patients (Gamma-Band Activity or GBA, between 40 and 150 Hz). Since then, an ever increasing number of studies have confirmed that GBA is a general index of neural processing in the human brain (provided some well-established guidelines to avoid any interpretation bias due to epilepsy) (Lachaux et al., 2012): in short, whenever a neural population gets recruited for a cognitive process, GBA recorded in its vicinity increases, for the duration of the process (Lachaux et al., 2012).

The main consequence of this discovery was that it was now possible to map the fine neural dynamics of human cognition with both millimetric and millisecond precision—a feat impossible so far. We argue that this discovery has profound influence on our understanding of human cognition in general, and on any attempt to combine neural and experiential micro-dynamics, because of four characteristics of this approach (intracranial Gamma-Band Mapping, or iGBM).

### Fine time resolution

iGBM measures neuronal activity rapidly enough (millisecond resolution) to enable the fast mental processes revealed by the elicitation method to be related to changes in the neural signal. This would not be possible with fMRI, for instance, whose time resolution is too slow to capture the subsecond microdynamics of experience, as it can occurs for instance during reading, or visual exploration, two processes paced by several saccades per second.

### Fine spatial resolution

iGBM measures neuronal activity with a spatial resolution equivalent to fMRI, a few cubic millimeters (Lachaux et al., 2003), which is roughly the grain of the modular organization of the human cortex. Within such volume, neural populations tend to share the same function (e.g., word-form recognition, music perception, etc.). In contrast, fast non-invasive measures such as Electroencephalography (EEG) and magnetoencephalography (MEG), have much lower spatial resolution and average together neural signals from regions with very heterogeneous functions, making it extremely difficult, if not impossible, to relate them to the precise cognitive operations revealed by experiential microdynamics. Of course, intracranial EEG recordings, and their high spatio-temporal resolution, can only be obtained in patients, mostly suffering from epilepsy (implantations in other neurological diseases are restricted to very specific brain regions). This is a limitation of every iEEG study and the “price to pay” for such recordings, which has been debated for years (see Lachaux et al., 2012). In a sense, the brain of those patients is always considered to be a model of normal, healthy, brains. The usual guideline is to present data that are reproducible across patients with different epileptogenic networks, and with normal or close to normal behavior (especially in cognitive tasks: performance close to normal). Following such guidelines, we have repeatedly reported functional networks in epileptic patients whose anatomical organization is fully compatible with fMRI data observed in normal subjects (Jung et al., 2008; Bastin et al., 2012).

### Single-trial precision level

Because intracranial electrodes record directly within neural structures, the signal-to-noise ratio of the signals does not need to be improved through averaging procedures (i.e., averaging signals recorded across multiple repetitions of the same cognitive situation), as is always the case with classic human brain measures. iGBM provides therefore a direct picture of the neural dynamics of singular, unique experiences, in single individuals. This is crucial for our project, whose objective is not to match generic experiential structures, in which the specificities of individual experiences would be erased, with generic neural signatures, in which deviations would be eliminated as noise, but singular experiences with their specific neural correlates. Of course, the goal is to detect patterns, but it is only in a second stage, from the analysis of singular experiences that generic structures are searched for at the experiential and neural levels.

In fact, the signal-to-noise ratio of iGBM signals is so good that it raises new problems in human cognitive neuroscience: the trial-to-trial variability it reveals in neural processing cannot be interpreted without finer information about the succession of cognitive operations the participant is performing, in each trial. We observed for instance that when participants have to remember a list of letters, neural processing in the working memory network is not continuous in single-trials (unpublished data). Averaging across trials averages out such discontinuity and gives an illusion that neural activity is maintained throughout the maintenance period, which is simply not true at the single-trial level. However, the experimental design itself does not provide sufficient information to understand the reasons for these discontinuities, which might simply be the coming-and-going of the memory trace. Only a fine analysis of the dynamics of the subject's experience could provide an answer. During reading also, single-trial analysis reveals that the activation of the phonological system (including the auditory cortex) by written words is far from systematic (Perrone-Bertolotti et al., 2012), while an average picture would hide that reality. Is it that participants do not systematically pronounce words mentally as they read them? Even when the phonological system is active, iGBM reveals neural processing in secondary auditory cortical areas specialized for speech-perception. However, is this the correlate of the phenomenal experience which dominates reading that is, the conversion of a written form into a mental sound? Only a detailed investigation of the first-person experiential dynamics of “what it is like to read,” at the single-trial level, could provide the answer. There are practical ways to do this; in the study above for instance, we observed a variable lag between neural processing in visual areas (first) and auditory areas (second), ranging between 400 and 800 ms. This raises the possibility that this lag might be correlated, on a trial-to-trial basis, with the phenomenal experience of a decoupling between the visual and auditory components of reading, with an actual report reflecting a variable time-lag between the two. Such new questions and hypotheses are extremely hard to tackle using standard experimental design and without a finer description of what the participant is actually doing and experiencing at the single trial level.

#### Brain tv

The unique properties of iGBM have been combined into a new system pushing the approach even further, BrainTV (Lachaux et al., 2007b; www.braintv.org). BrainTV is simply a device for analyzing iEEG signals in real-time to provide feedback to the participants and experimenters about the amount of neural processing performed by a focal brain region, online. The region is freely chosen among all implanted cortical sites (typically 100–200), which can include high-order sensory, motor, limbic, associative, or executive cortex. The novelty of the approach is twofold: first, the fine dynamics of neural processing can be monitored in real-life situations, as the patient freely interacts with his environment, in sharp contrast with the strong constraints of neuroimaging; second, patients can actively participate in the research process, joining the research team to identify correlations between their brain activity and fine, private aspects of their ongoing experience, invisible to the experimenters. One important feature of BrainTV is that it continuously displays the time-course of neural activity over the last 10 s, so that participants are not required to divide their attention between the display and their mental processes. The investigation is sequential rather than parallel. BrainTV was recently used in an experiment requiring a patient to perform mental imagery (Hamamé et al., 2012). There is no such thing as mental imagery—the ability to create and maintain a mental visual image—without phenomenal experience. Therefore, how is it possible to identify the neural correlates of mental imagery, given that this phenomenal experience is accessible to the subject only, by definition? BrainTV solves that problem by providing the subject with an immediate feedback of his brain activity (as an image or as a sound). This provides a direct way to combine first-person, subjective and third person, objective data in one single *locus*, the subject's mind, and allow a comparison process, to detect possible correlations. This comparison task, which requires that the subject has a sharp awareness of the dynamics of his experience, is in our approach highly facilitated by the use of elicitation interviews, which enable even untrained subjects to acquire fine-grained consciousness of their experience during a given slice of time. In a sense, BrainTV shares similarities with real-time fMRI, considering recent evidence by our group and others (see Lachaux et al., 2007a) that the BOLD signal is strongly correlated with gamma-band activity. The main difference is obviously that fMRI can be used to monitor any brain region in healthy subjects, including meditators (see Garrison et al., 2013) who are trained in introspecting. While iGBM can only be applied in patients, but with a much higher time-resolution and better signal-to-noise ratio, the lack of introspective skill being counterbalanced by the use of interviews. We believe that the two approaches are complementary and should mutually enlighten each other in the future (Lachaux et al., 2007b).

One characteristic of BrainTV is that it not only enables the subject to identify the neural correlates of subtle variations of the phenomenal aspects of a mental image (such as its intensity or localization); but also, by giving him a real time picture of his brain microdynamics, it draws his attention toward subtle modulations of his experience he is not usually aware of. We used that approach to show that Gamma-Band Activity in the Inferior Temporal Gyrus is highly correlated with the vividness of a mental image (Hamamé et al., 2012). The next step is to provide in addition online measures of functional connectivity, considering the acknowledged importance of long-distance neural interactions for cognition (Varela et al., 2001). We are currently conducting the first studies of that kind, with a measure of connectivity based on amplitude-amplitude coupling in the gamma range, following recent (offline) evidence that such coupling is modulated by cognitive activity (Vidal et al., 2012).

One critical question is whether participants can find traces in their subjective experience of any change in neural activity (as quantified by local GBA or long-range GBA correlations), recorded in any cortical region. Are there regions “hidden” to subjective experience? Only elicitation sessions designed to probe the correlation between subjective experience and the fluctuations of neural activity can answer that long-lasting question, directly related to the issue of the neural correlates of consciousness.

## Conclusion

New methods enable us to collect descriptions of neural and experiential dynamics at a fine level of granularity that seems to be the right level to search for correlations. By unfolding these microdynamics, these methods provide access to early and usually invisible stages of our cognitive processes, where the distinction between the sensorial modalities, and between the “subject” and “object” poles seems to be less rigid than in later stages. We hypothesize that these early stages give us a glimpse on the process of co-constitution of subject and object, knower and known that is called “enaction” (Varela et al., 1991; Lutz and Thompson, 2003). Therefore, the twofold microdynamic approach that we are advocating would not only provide a methodological solution to the problems of correlation between experiential and neuronal, first-person and third-person descriptions of our cognitive processes. It would also open a line of investigation into the very cognitive acts in which the scission between the objective and the subjective worlds originates, and a means to verify and refine the dynamic epistemology of enaction.

### Conflict of interest statement

The authors declare that the research was conducted in the absence of any commercial or financial relationships that could be construed as a potential conflict of interest.

## Footnotes

^1^Unlike, for example, temperature and texture, which are specific to touch, or color, which is specific to sight.
